# PHD3 regulates differentiation, tumour growth and angiogenesis in pancreatic cancer

**DOI:** 10.1038/sj.bjc.6605936

**Published:** 2010-10-26

**Authors:** Y Su, M Loos, N Giese, O J Hines, I Diebold, A Görlach, E Metzen, S Pastorekova, H Friess, P Büchler

**Affiliations:** 1 Department of General Surgery, University of Heidelberg, Im Neuenheimer Feld 110, Heidelberg 69120, Germany; 2 Department of Surgery, Zhongda Hospital, Nanjng Southeast University, Nanjing 210009, P.R. China; 3 Department of General Surgery, Klinikum rechts der Isar, Technische Universität München, Ismaninger Strasse 22, München 81675, Germany; 4 Department of Surgery, UCLA School of Medicine, University of California, Los Angeles, CA 90095, USA; 5 Department of Paediatric Cardiology and Congenital Heart Disease, Experimental and Molecular Paediatric Cardiology, German Heart Center Munich at Technische Universität München, Munich 80636, Germany; 6 Institute of Physiology, University Hospital of Essen, University of Duisburg-Essen, Hufelandstraße 55, Essen 45147, Germany; 7 Institute of Institute of Virology, Centre of Molecular Medicine, Slovak Academy of Sciences, Dubravska cesta 9, Bratislava 84505, Slovak Republic

**Keywords:** angiogenesis, HIF-1, hypoxia, pancreatic cancer, PHD3

## Abstract

**Purpose:**

Tumour hypoxia activates hypoxia-inducible factor-1 (HIF-1) and indluences angiogenesis, cell survival and invasion. Prolyl hydroxylase-3 (PHD3) regulates degradation of HIF-1*α*. The effects of PHD3 in tumour growth are largely unknown.

**Experimental design:**

PHD3 expression was analysed in human pancreatic cancer tissues and cancer cell lines by real-time quantitative PCR and immunohistochemistry. PHD3 overexpression was established by stable transfection and downregulation by short interfering RNA technology. VEGF was quantified by enzyme-linked immunosorbent assay. Matrigel invasion assays were performed to examine tumour cell invasion. Apoptosis was measured by annexin-V staining and caspase-3 assays. The effect of PHD3 on tumour growth *in vivo* was evaluated in an established orthotopic murine model.

**Results:**

PHD3 was upregulated in well-differentiated human tumours and cell lines, and regulated hypoxic VEGF secretion. PHD3 overexpression mediated tumour cell growth and invasion by induction of apoptosis in a nerve growth factor-dependent manner by the activation of caspase-3 and phosphorylation of focal adhesion kinase HIF-1 independently. *In vivo*, PHD3 inhibited tumour growth by abrogation of tumour angiogenesis.

**Conclusion:**

Our results indicate essential functions of PHD3 in tumour growth, apoptosis and angiogenesis and through HIF-1-dependent and HIF-1-independent pathways.

Pancreatic cancer is one of the most lethal human malignancies for still unknown reasons (Jemal *et al*, 2007). Tumour hypoxia has become an area of special interest as it has been correlated with therapeutic resistance and increased invasion, metastasis and poor outcome (Graeber *et al*, 1994; Buchler *et al*, 2004; Vaupel *et al*, 2005). Within this context the transcription factor Hypoxia-inducible factor-1 (HIF-1) has been identified as a major regulator of oxygen homoeostasis (Wang and Semenza, 1995; Wang *et al*, 1995). HIF-1 consists of two subunits: the hypoxia sensitive HIF-1*α* subunit and the constitutively expressed HIF-1*β* subunit (Wang and Semenza, 1995; Wang *et al*, 1995). Under normoxic conditions HIF-1*α* is subject to rapid degradation by means of hydroxylation of specific proline residues that enables binding of the von Hippel–Lindau protein and the formation of an E3 ubiquitin ligase complex that targets HIF-*α* for proteosomal degradation (Semenza, 2007).

Three prolyl hydroxylase domain containing proteins, PHD1, PHD2 and PHD3 (PHDs, HIF-PHDs or Egl-9 homologues) mediate oxygen-dependent degradation of HIF-1*α* through von Hippel–Lindau protein (Epstein *et al*, 2001; Metzen and Ratcliffe, 2004). All three PHDs catalyse site-specific hydroxylation of HIF-*α* at Pro-564. Under hypoxic conditions PHDs show reduced enzyme activity resulting in stabilisation of HIF-1*α*. The expression of PHDs is regulated by a variety of stimuli, including hypoxia, which induced PHD2 and PHD3 expression (Epstein *et al*, 2001; Bruegge *et al*, 2007). Hypoxia-induced PHD3 expression results in enhanced rates of HIF-1*α* degradation. It is interesting that PHD3 appears to be a direct target of HIF-1 serving as a negative feedback mechanism (Marxsen *et al*, 2004). However, the physiological role is not well understood.

Only little is known about PHD3 in human cancer growth. In the case of pancreatic cancer a recent report found that PHD3 is overexpressed in pancreatic cancer and might influence patient's survival (Metzen and Ratcliffe, 2004; Bruegge *et al*, 2007; Gossage *et al*, 2010). In the present study, we focus on the regulatory function of PHD3 in tumour growth and progression, including its role in apoptosis and angiogenesis *in vitro* and *in vivo*.

## Patients and methods

### Patients

Human pancreatic tissue specimens were obtained from 62 patients who underwent resection for ductal adenocarcinoma of the pancreas and 20 organ donors, where no recipients for transplantation were present. The study protocol was approved by the Ethics Committee of the University of Heidelberg, Germany.

### Cell culture

Pancreatic cancer cell lines Capan-1, Capan-2, MIA PaCa-2 and PANC-1 were obtained from the American Tissue Type Culture Collection (Rockville, MD, USA) and cultured as described (Buchler *et al*, 2005). For hypoxia studies a modular incubator chamber was flushed for 20 min with a gas mixture consisting of 0.75% O_2_, 10% CO_2_ and 89.25% N_2_. Recombinant human nerve growth factor (NGF) was purchased from R&D Systems (Minneapolis, MN, USA). Hydroxylase inhibitor dimethyloxaloylglycine (DMOG) was obtained from Biomol GmbH (Hamburg, Germany) and used at 40 *μ*M. 3-(5′-hydroxymethyl-2′-furyl)-1-benzyl indazole (YC-1) was purchased from AG Scientific Inc. (San Diego, CA, USA), resuspended in DMSO at a stock concentration of 120 mg ml^−1^, and stored at −30°C. YC-1 was added at a concentration of 30 *μ*M 1 h before the assay. The concentration of 30 *μ*M YC-1 was chosen on the basis of the previous experiments, in which hypoxic accumulation of HIF-1*α* protein levels were reduced by >70% in both cell lines investigated (Supplementary Figure).

### RNA preparation and real-time quantitative PCR qRT-PCR

All reagents and equipment for mRNA and cDNA preparation were purchased from Roche Applied Science (Mannheim, Germany). The mRNA was isolated by automated MagNA Pure LC instrument and cDNA was generated. RT-PCR was performed with the LightCycler FastStart DNA SYBR Green kit as described previously (Buchler *et al*, 2005). The number of specific transcripts was calculated from the standard curve of two housekeeping genes, *cyclophilin B* and *hypoxanthine phosphoribosyltransferase*. The data of two independent analyses for each sample and parameter were averaged and presented as adjusted transcripts per *μ*l of cDNA.

### Generation of PHD3-overexpressing PANC-1 and MIA PaCa-2 cells

PANC-1 and MIA PaCa-2 cells were converted into PHD3-overexpressing cells by stable transfection of the pEGFP-N1 (Clontech, Palo Alto, CA, USA) expression plasmid carrying full-length PHD3 cDNA using the TransFectin Lipid Reagent (Bio-Rad Laboratories, München, Germany). Selection was done in G418 containing culture medium resulting in a pool of transfected cells. Empty pEGFP-N1 plasmids were transfected as a negative control.

### Short interfering RNA (siRNA) treatment

Cells were less than 50% confluent and transfected with 5.0 nM siPHD3 at 24 and 48 h using Oligofectamine (Invitrogen, Darmstadt, Germany) according to the manufacturer’s instructions. Sequences of PHD3 siRNA were (sense: 5′-GUACUUUGAUGCUGAAGAAUU-3′ antisense: 5′-UUCUUCAGCAUCAAAGUACUU-3′, Genebank accession number: EGLN3NM_022073) and luciferase (siControl scrambled Non-Targeting siRNA) purchased from Applied Biosystems (Applera Deutschland GmbH, Darmstadt, Germany).

### Apoptosis measurement and caspase-3 activity assays

For annexin-V-binding studies an annexin-V-FLOUS Staining Kit (Boehringer Mannheim GmbH, Mannheim, Germany) was used as previously described in detail (Buchler *et al*, 2003a). Annexin-V-positive and propidium-iodide-positive cells were considered as necrotic cells, whereas annexin-V-positive and propidium-iodide-negative cells were counted as apoptotic cells. Details of the caspase assays were also previously described (Buchler *et al*, 2003a). Data were expressed as mol AMC permg protein per minute and given as means±s.e. for at least three preparations. Caspase-3 substrate and caspase-3 inhibitor Z-Val-Ala-Asp-fluoromethylketone were purchased from Calbiochem (San Diego, CA, USA).

### Protein studies

Western blot analyses were carried out as previously described (Buchler *et al*, 2003a). Briefly a mouse monoclonal anti-HIF-1*α* (diluted 1 : 750, Transduction Laboratories, San Diego, CA, USA), a mouse monoclonal anti-*α*-tubulin (diluted 1 : 750, Santa Cruz Biotechnology, Heidelberg, Germany), a rabbit polyclonal anti-human PHD3 antibody (diluted 1 : 200; AB4562, Abcam, Cambridge, UK) were used. Polyclonal antibodies to focal adhesion kinase (FAK) and to the phosphoprotein p-FAK were from Santa Cruz Biotechnology.

Immunohistochemistry was done manually as previously described (Buchler *et al*, 2003b). The rabbit polyclonal anti-human PHD3 antibody (diluted 1 : 200; AB4562, Abcam), the mouse monoclonal anti-HIF-1*α* (diluted 1 : 100 Transduction Laboratories) and for carbonic anhydrases IX (CAIXs) a murine monoclonal antibody M75 recognising the N-terminal domain of CAIX in dilution of 1 : 250 were used as primary antibodies. Tissues from normal human pancreas and human pancreatic cancer were fixed in formalin and embedded in paraffin. Tissue sections (5 *μ*m) were deparaffinised and rehydrated. After deparaffinisation and rehydration antigen retrieval was performed by cooking in 0.1 mol l^−1^ sodium citrate buffer for 25 min, followed by treatment with 3% hydrogen peroxide solution to block endogenous peroxidase activity. Incubation with primary antibodies was carried out over night at 4°C. After standard washing steps slides were subsequently incubated with a biotinylated secondary antibody and streptavidin-peroxidase. Slides were exposed to 3.3′-diaminobenzidine tetrahydrochloride (Dako GmbH, Hamburg, Germany) for colour development and counterstained with hematoxylin.

Quantification of immunohistochemical staining was done by using a scale ranging from 0 to 3 dependent upon the number of positively stained (tumour) cells. PHD3 expression was analysed according to the percentage of cells showing cytoplasmic positivity. The grading was done as following: grade 0, 0–10% grade 1+, 10–20% grade 2+, 20–50% and grade 3+, >50%. The degree of monoclonal antibody reactivity in individual tissue sections was considered positive if unequivocal staining of the cytoplasm was seen in more than score 2+. The slides were analysed independently by two investigators blinded to the underlying clinicopathological condition.

Determination of VEGF protein levels in cell supernatant was done as described (El Fitori *et al*, 2007). MIA PaCa-2 and PANC-1 cells were seeded at 2.5 × 10^6^ cells per 100 mm tissue culture plate and equal densities for each cell line were analysed. Cells were grown for 24 h in DMEM medium supplemented with 10% FBS, washed three times with PBS and changed to exactly 10 ml of DMEM medium supplemented with 1% FBS. Identical cultures for normoxic and hypoxic culture were analysed. Cells were harvested for protein analysis and cell count. As VEGF is a secreted protein the amount of VEGF protein in the supernatant of cells was determined with an enzyme-linked immunosorbent assay kit (R&D Systems) according to the manufacturer’s instructions. The VEGF protein levels was expressed as per gram of VEGF protein per 10^5^ cells.

### Invasion assay

Cell invasion was studied using the BioCoat Matrigel Invasion Chambers (Becton Dickinson, Bedford, MA, USA). 2.5 × 10^4^ cells were seeded into the upper chamber of the invasion chambers and incubated for 24 h for consolidation after seeding. For hypoxia studies (48 h), the BioCoat Matrigel invasion chamber was placed in the hypoxic chamber that was flushed for 20 min with a gas mixture consisting of 0.75% O_2_, 10% CO_2_ and 89.25% N_2_. Cells adherent to the lower surface were fixed in 75% methanol mixed with 25% acetone and then stained with 1% Toluidine blue. Cells were counted under a microscope at × 200 magnification. The invasion index was expressed as the ratio between the numbers of invaded test cells to the number of invaded control cells.

### Orthotopic pancreatic cancer model

An orthotopic metastatic nude mouse (BALB/cA) model was used as described (Fu *et al*, 1992; Buchler *et al*, 2007). After 6 weeks animals underwent autopsies. Metastatic tumour spread was determined macroscopically in all thoracic, abdominal, retroperitoneal and pelvic organs, and all suspicious lesions were confirmed by microscopic analysis. Metastatic spread was quantified by counting the different organs, which contained metastatic lesions. Thus, every point in the metastatic score represented a different organ of metastatic tumour spread (Buchler *et al*, 2004). The tumour volume was calculated as described previously using the following formula: tumour volume=0.5 × (length × width × depth) (El Fitori *et al*, 2007).

### Assessment of microvessel density in xenograft tumours

For assessment of vascularity, frozen sections, each 5 *μ*m thick, were cut and immunostaining of the endothelial cells were done with anti-mouse CD31 antibody (BD Pharmingen, CA, USA) as previously described (Buchler *et al*, 2007). Stained tissue specimens were analysed by two independent observers unaware of the animal’s status. Microvessel density was determined as described by Vermeulen (Vermeulen *et al*, 2002).

### Statistical analysis

Experiments were done in triplicates and repeated at least two times. Results are expressed as mean±s.e. Statistical significance was determined by Student's *t*-test (*P*<0.05).

## Results

### PHD3 is upregulated in human pancreatic cancer samples

**Figure 1:**
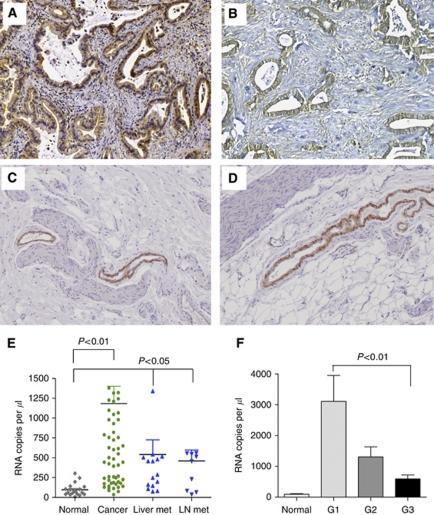
PHD3 expression in human pancreatic cancer tissue specimens: PHD3 immunohistochemistry of pancreatic ductal adenocarcinoma specimens (**A**–**D**). qRT-PCR (**E**) of normal human pancreatic tissue and tissue specimens of primary tumours, liver and lymph node metastases (**E**). Correlation of PHD3 mRNA expression levels with tumour grading (**F**). *P*<0.05 and *P*<0.01 indicates statistical significance.

Localisation of PHD3 expression in human tissue specimens was analysed by immunohistochemistry. In normal pancreatic tissue sections immunoreactivity for PHD3 was present in pancreatic acini and pancreatic islet cells (not shown). In contrast, pancreatic cancer tissues exhibited in well-differentiated specimens a strong PHD3 immunoreactivity in ductal cancer cells in which a primarily cytoplasmic staining pattern was present (Figure 1A–D). Strong immunoreactivity was also observed in cancer cells approaching nerves within the tumour without histological signs of tumour invasion into these nerves (Figure 1C and D). Overall there was a clear trend that better-differentiated tumours exhibited a stronger PHD3-staining intensity when compared with less-differentiated tumour specimens. A possible relationship between PHD3 expression, HIF-1 activation and CAIX activation was assayed using serial slides. There was a regular co-localisation of HIF-1 and CAIX, but no clear correlation was detectable with PHD3 (not shown).

PHD3 mRNA expression was determined in 20 normal pancreatic tissue samples, 62 pancreatic cancer tissue samples, 16 specimens of liver metastases and 10 lymph-node metastases of pancreatic cancer. (Figure 1E). In comparison to normal pancreatic tissues PHD3 mRNA expression was significantly elevated in pancreatic cancer tissues (*P*<0.01). The average replicate number of PHD3 mRNA was 96±76 in normal pancreas. In contrast, a more than 10-fold increased PHD3 mRNA expression level was found in pancreatic cancer specimens with an average replicate number of 1183±219 mRNA copies per *μ*l (Figure 1E). Similar to the observation in immunohistochemical analysis, there was a clear trend to higher PHD3 mRNA levels in more-differentiated tumour specimens. Well-differentiated tumour samples had significantly higher PHD3 mRNA levels when compared with undifferentiated tumour samples (*P*<0.0001) (Figure 1F). Similarly metastatic lesions as found in liver metastases and lymph nodes expressed higher levels of PHD3 mRNA (*P*<0.05), when compared with normal pancreatic tissue (Figure 1E).

### PHD3 mRNA expression in cultured pancreatic cancer cell lines

**Figure 2:**
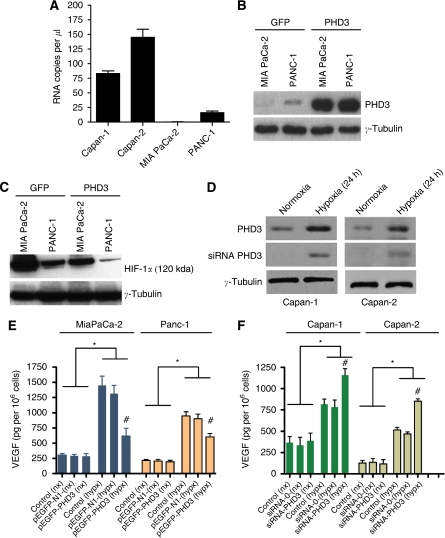
Modulation of PHD3 expression in human pancreatic cancer cell lines and hypoxic gene expression: PHD3 mRNA levels in pancreatic cancer cell lines were measured by qRT-PCR (**A**). PHD3 transfection increased PHD3 protein levels in MIA PaCa-2 and PANC-1 cells (**B**). HIF-1 western blot analysis of PHD3 and control tranfected cells after 16 h of hypoxia (**C**). Targeting PHD3 expression by siRNA. PHD3 western blot of siPHD3 or control siRNA-treated Capan-1 and Capan-2 cells (**D**). Whole-cell extracts were resolved on 10% SDS polyacrylamide gels, proteins were transferred to nitrocellulose membranes and probed with antibodies against PHD3. Incubation with the *γ*-tubulin monoclonal antibody was performed as a loading control (**B**, **D**). VEGF secretion determined by enzyme-linked immunosorbent assay (**E**). Capan-1 and -2 cells were treated by siRNA to suppress PHD3 expression, whereas MIA PaCa-2 and PANC-1 cells were transfected with a PHD3 expression vector to restore PHD3 expression (**F**). ^*^P<0.05 indicates statistical significance of hypoxic *vs* normoxic treatment; ^#^*P*<0.05 indicates statistical significance within the hypoxic treatment panel.

PHD3 mRNA expression in cultured pancreatic cancer cell lines Capan-1, PANC-1 and MIA PaCa-2 was also determined by qRT-PCR. PHD3 mRNA expression was detected in the well-differentiated tumour cell lines Capan-1 (83±8copies per *μ*l) and Capan-2 (145±24copies/*μ*l). The less-differentiated PANC-1 cell line expressed only low levels of PHD3 mRNA (16±3copies per *μ*l), whereas the undifferentiated MIA PaCa-2 cell line was entirely devoid of PHD3 mRNA expression (Figure 2A).

### PHD3 directly modulates hypoxic HIF-1 target gene expression

PHD3 has been shown to catalyze proteolytic degradation HIF-1*α* and inhibition of VEGF gene expression. As pancreatic cancer grows under hypoxic conditions, it was tested whether PHD3 overexpression influences HIF-1*α* stabilisation and VEGF secretion. For this purpose PHD3 expression was induced in cell lines with low or absent PHD3 expression PHD3 (MIA PaCa-2 and PANC-1). Both cell lines were stably transfected either with a PHD3 expression vector or an empty pEGFP/N1 vector for control experiments. Upon cell transfection, PHD3 expression was stably induced in both cell lines (Figure 2B). The functional relevance was tested by exposing cells to hypoxia for 16 h. HIF-1*α* protein was downregulated by PHD3 overexpression under hypoxic conditions but, nevertheless, HIF-1*α* protein was still detectable at low levels (Figure 2C). Conversely, Capan-1 and Capan-2 cells expressed high PHD3 mRNA levels constitutively. Therefore, experimental downregulation of PHD3 was achieved by using specific siRNAs directed against PHD3. Upon siRNA treatment, PHD3 protein was not detectable by western blot analysis under normoxia in Capan-1 and -2 cells, but a slight protein band was detectable under hypoxic conditions. To test whether HIF-1 target gene expression was affected by modulation of PHD3 expression we assessed VEGF protein levels (Figure 2E and F). Under normoxic conditions PHD regulation had no effect on VEGF secretion (Figure 2E and F). Hypoxic culture conditions itself increased VEGF protein secretion in all cell lines. Overexpression of PHD3 in MIA PaCa-2 and PANC-1 cells significantly reverted hypoxic induction of VEGF secretion (*P*<0.05). Reversely downregulation of PHD3 by siRNA in Capan-1 and -2 cells expressing PHD3 resulted in a marked increase of VEGF secretion when compared with mock transfected cells (Figure 2F).

### PHD3 modulates tumour cell growth, invasion and cell morphology

**Figure 3:**
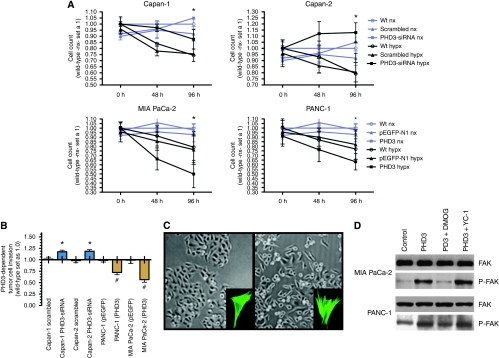
HD3 negatively regulates pancreatic cancer growth and tumour cell invasion: (**A**) Cells were cultured under regular culture conditions (nx; blue lines) or conditions of hypoxia (hypx; black line) for the indicated time. Cell count was done as described and untreated normoxic wild-type (wt) cells were considered as reference and set as 1.0. Knockdown of PHD3 expression in Capan-1 and -2 was achieved by siRNA against PHD3 sequences and compared with scrambled siRNA sequences or untreated (wt) cells. Overexpression of PHD3 in MIA PaCa-2 and PANC-1 cells was achieved by cell transfection with a full-length PHD3 cDNA cloned into a pEGFP/N1 expression vector (**A**). (**B**) Matrigel-based invasion assay. Pancreatic cancer cells were grown for 48 h under hypoxic culture conditions and the assay performed and calculated as described in Materials and methods. Wild-type cells were set as 1.0 and served as reference. (**C**) Upon cell transfection, the cell shape of MIA PaCa-2 cells changed with a more scattered cell growth and some cells detaching from the surface (**C**). Phase contrast images of PHD3 overexpressing MIA PaCa-2 cells showing clustered morphology in the control group (**C** left) and scattered cell growth in the PHD3 expressing cells (**C** right). These images were acquired with a × 10 objective. The insert in C shows stress fibre formation after PHD3 overexpression. Human MIA PaCa-2 cells were seeded on chamber slides (10 000 cells/well) MIA PaCa-2 wild-type cells (left insert) and PHD3-transfected cells 24 h after cell transfection (right insert). Cells were fixed, permeabilised, and stained with Alexa Fluor 488 phalloidin, which shows F-actin sites. (**D**) Effect of PHD3 on FAK phosphorylation in MIA PaCa-2 and PANC-1 cells. Cells were cultured for 24 h in DMEM supplemented with 0.5% FBS and the indicated inhibitors for PHD3 (dimethyloxaloylglycine) and HIF1*α* (YC-1) were added and then cells grown for an additional 12 h in serum-free medium. Cell lysates were prepared and subjected to immunoblot analysis with antibodies to FAK or to phosphorylated FAK (p-FAK). ^*^ or ^#^ indicates *P*<0.05 when compared with wt cells.

Uniformely hypoxic cell culture conditions *per se* led to growth retardation in all cell lines (Figure 3A). Knockdown of PHD3 using siRNA against PHD3 caused an increase in cell number under hypoxia in Capan-1 and Capan-2 cells when compared with cells treated with scrambled siRNAs or wild-type cells (Figure 3A). Overexpression of PHD3 in MIA PaCa-2 and PANC-1 cells significantly accelerated growth suppression under hypoxic conditions when compared with wild-type or mock-transfected (empty pEGFP/N1 vector) cells (Figure 3A). The tendency that PHD3 expression exhibited growth-suppressive effects was seen under conditions of normoxia as well (Figure 3A). Whether these observations were because of the HIF-1 activation was tested by using the HIF-1 inhibitor YC-1, but no clear effect of YC-1 on cell growth was detectable (data not shown). In Matrigel-based invasion assays overexpression of PHD3 in MIA PaCa-2 and PANC-1 cells significantly reduced tumour cell invasion (*P*<0.05). In the case of PANC-1 a 27.5% reduction of cell invasion was observed and in the case of MIA PaCa-2 invasion was reduced by 43.7% (Figure 3B). Furthermore, the PHD3-transfected MIA PaCa-2 and PANC-1 cells exhibited a clear change in cell morphology and growth pattern, showing a more scattered and dispersed cell-growth pattern when compared with controls (Figure 3C). On the basis of this observation we tested whether changes in cell growth also led to changes in cell's contractility, as enhanced contractility and formation of stress fibres are required for cell migration and matrigel invasion. Visualisation of stress fibres was achieved by Alexa Fluor 488 phalloidin incorporation after transfection of MIA PaCa-2 and PANC-1 cells. PHD3 transfection induced the formation of stretched bundles of stress fibres when compared with control cells (Figure 3C). Because FAK activity has a key role in actin polymerisation and stress fibre formation, we tested the effect of PHD3 overexpression on FAK phosphorylation in MIA PaCa-2 and PANC-1 cells. Upon PHD3 overexpression there was a clear increase in phospho-FAK, but no increase in total FAK was detectable (Figure 3D). The effect required PHD3 hydroxylase activity, as DMOG treatment abrogated FAK phosphorylation. DMOG is a well-characterised PHD inhibitor (DMOG) used in a concentration of 40 *μ*M (Biomol GmbH). In contrast, FAK phosphorylation was seen independent of HIF-1, as the HIF-1 inhibitor YC-1 did not affect FAK phosporylation (Figure 3D).

### PHD3-induced NGF-dependent apoptosis by activation of caspase-3 activity

**Figure 4:**
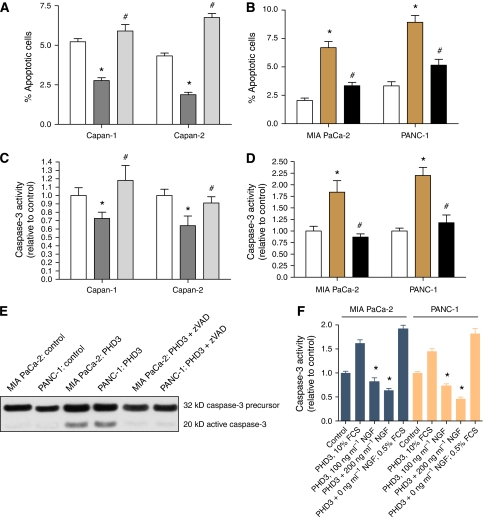
Effects of a PHD3 overexpression on apoptosis in pancreatic cancer cells. Effects of modulation of PHD3 expression on apoptosis (**A** and **B**) and caspase-3 activation (**C**–**F**). In **A** and **B** Annexin-V staining was used to quantify apoptosis and in **C** and **D** caspase-3 activity was assayed as described in methods. The clear bar in panel **A** and **C** shows the results of native Capan-1 and -2 cells. The dark bar represents siRNA PHD3 experiments and the grey bar cells treated with scrambled siRNA moieties. In panel **B** and **D** the clear bar represents cells transfected with the empty vector (pEGFP), the grey bar reflects the results obtained by PHD3 overexpression and the black bar reflects the results obtained of PHD3-transfected cells treated with dimethyloxaloylglycine for 24 h before analysis. (**E**) MIA PaCa-2 and PANC-1 cells were cultured for 72 h. Cell lysates were obtained and subjected to western blot analysis. Activation of caspase-3 was manifested by the decrease in pro-caspase-3 (32 kD) and concomitant increase in active caspase-3 fragment (20 kD). The pan-caspase inhibitor Z-Val-Ala-Asp-fluoromethylketone (50 *μ*M, +Z-VAD) was used to inhibit caspase-3. In panel **F** experiments were performed as described in 35-mm diameter dishes. 3 × 10^4^ cells per 35-mm dish were inoculated in 2 ml of medium containing 10% FCS. After 24 h, cells were washed twice with serum-free medium. Next day, the medium was replaced with 2 ml of 0.5% serum containing medium with addition of NGF at the indicated doses. Values represent means±s.e. for at least three independent experiments. ^*^P<0.05 indicated statistically significant differences compared with control-treated cells. ^#^*P*<0.05 indicates statistically significant differences in comparison to PHD3-modulated cells.

The morphology of cells overexpression PHD3 was quite distinct (Figure 3C) and it appeared that some of these cells undergo apoptosis. Therefore, one likely reason for reduced tumour cell invasion might be the induction of apoptosis (Figure 4A–F). Upon PHD3 expression apoptosis increased in MIA PaCa-2 and PANC-1 cells sharply (Figure 4B). Treatment with DMOG reverted the proapoptotic effect of PHD3 expression almost completely in PANC-1 and MIA PaCa-2 cells suggesting that apoptosis required PHD3 activity. In order to confirm that PHD3 expression regulates apoptosis siRNA experiments were performed. Suppression of PHD3 expression in Capan-1 and Capan-2, led to a decrease in apoptosis in these cell lines (Figure 4A). These findings suggest that PHD3 has growth-suppressive and anti-invasive properties triggered by the induction of apoptosis. This process was seen independently of HIF-1, as YC-1 treatment did not affect apoptosis in these cell lines (data not shown). The mechanism by which apoptosis is mediated was further analysed by caspase-3 assays, as caspase-3 activation is involved in apoptosis in pancreatic cancer cells (Buchler *et al*, 2003a). Therefore, we analysed whether PHD3 activity is linked to activation of caspase-3 and found that PHD3 overexpression increased caspase-3 activity (Figure 4D). Further evidence that caspase-3 activity is controlled by PHD3 was gained in siRNA experiments suppressing constitutive PHD3 activity in Capan-1 and -2 cell lines, in which caspase-3 activity was inhibited upon PHD3 suppression (Figure 4C). PHD3 increased caspase-3 activity as seen by the appearance of the cleaved 20 kD active caspase fragment (Figure 4E). Activation of caspase-3 was inhibited by Z-Val-Ala-Asp-fluoromethylketone, a broad-spectrum caspase inhibitor, which blocked PHD3-induced caspase activation (Figure 4E). Similarly caspase-3 activation was not altered in cells treated with YC-1 suggesting that the effect of PHD3 on apoptosis was independent of HIF-1 (data not shown).

Because of the *in vivo* observation that PHD3 immunoreactivity was strongly stained next to nerves in pancreatic cancer we determined the role of NGF in PHD3-mediated apoptosis, as previous reports indicated that NGF influences apoptosis (Lee *et al*, 2005). NGF has previously been shown to be a direct mitogenic factor in pancreatic cancer and presumably influences perineural cancer invasion and metastatic spread (Zhu *et al*, 1999). PHD3-induced apoptosis was suppressed by addition of increasing doses of NGF with the maximum survival observed at 200 ng ml^−1^ (Figure 4F). Serum starvation and NGF withdrawal with PHD3 overexpression caused the highest apoptotic rate in both MIA PaCa-2 and PANC-1 cells, suggesting that NGF suppresses PHD3-triggered apoptosis. To test whether this effect was specific for NGF and not a general phenomenon among growth factors we also tested whether VEGF (10 ng ml^−1^), FGF-2 (5 ng ml^−1^) or PDGF-AB (20 ng ml^−1^) and found no effect of caspase-3 activity (data not shown).

### *In vivo*, PHD3 regulates tumour growth and angiogenesis in an orthotopic mouse model

**Figure 5:**
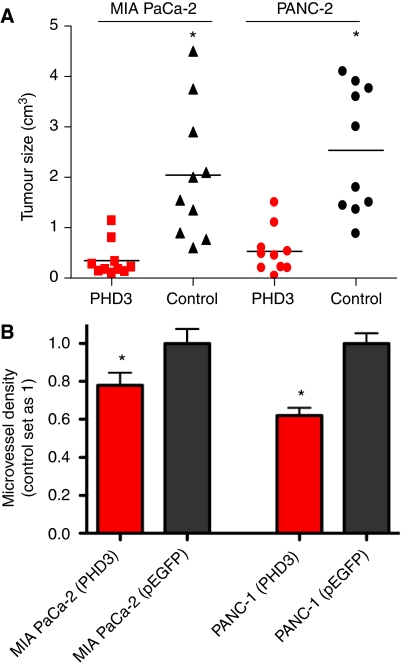
Tumour growth in an orthotopic mouse model of pancreatic cancer and microvessel density analysis: Xenograft tumours were induced by transplantation of small tumour fragments derived from a subcutaneous tumour into the pancreas of nude mice. Stable transfected MIA PaCa-2 and PANC-1 cell transfected with a PHD3 expression vector or with an empty pEGFP/N1 were used for subcutaneous tumour induction. After 6 weeks of tumour growth, PHD3 overexpressing tumours were significantly smaller than pEGFP/N1-transfected tumours (**A**). (**B**) Microvessel density of tumour xenografts from PANC-1 and MIA PaCa-2 cancer cells was evaluated by immunostaining for anti-CD31 antibody. Control xenograft tumours were set as 1. PHD3-overexpressing tumours showed a significant decrease in microvessel density compared with xenograft tumours derived from control-transfected tumour cells (**B**). ^*^ indicates *P*<0.05.

To determine the effect of PHD3 overexpression on tumour growth and angiogenesis *in vivo*, we used an orthotopic mouse model. A pool of selected PHD3 overexpressing MIA PaCa-2 and PANC-1 cells were injected into donor mice for subcutaneous tumour formation. After a 4-week period of subcutaneous tumour formation, one small tumour fragment was used for orthotopic tumour transplantation into the tail of the mouse pancreas. Around 6 weeks after tumour orthotopic tumour induction, mice were killed. Recombinant overexpression of PHD3 inhibited pancreatic cancer growth in nude mice substantially (Figure 5A), as the average sizes of PHD3 overexpressing tumours was significantly smaller when compared with control xeonograft tumours (*P*<0.05). The growth inhibitory activity of PHD3 was seen in both cell lines tested (Figure 5A). First, we tested whether growth inhibition was due to increased apoptosis in PHD3-overexpressing xenografts. Tunel staining did not reveal any difference between the different xenografted cells. As PHD3 inhibited VEGF production *in vitro* we assessed microvessel density of the harvested tumour xenografts (Figure 5B) and found a markedly decreased microvessel density in those xenograft tumours derived from PHD3-overexpressing cell clones (Figure 5B). PHD3 overexpression reduced metastatic tumour growth in mice bearing MIA PaCa-2 xenograft tumours with a metastatic score of 0.68±0.17 *vs* 1.4±0.25. No significant difference was seen in the metastatic score of PANC-1 xenografted tumours.

## Discussion

Hypoxia is a key microenvironmental condition in growth and metastasis of pancreatic cancer with HIF as the key molecular player in the process of adaptation to low oxygen levels (Rankin and Giaccia, 2008). HIF is a heterodimer of a constitutively expressed HIF-1*β* subunit, and one of three oxygen-regulated HIF-*α* subunits (HIF-1*α*, HIF-2*α* or HIF-3*α*). HIF activation is a multi-step process involving HIF-*α* stabilisation, nuclear translocation, heterodimerisation and transcriptional activation (Pouyssegur *et al*, 2006). However, HIF does not directly sense variations in oxygen tension. Instead, there are two types of oxygen sensors that control HIF action: The PHD domain proteins (PHD1-3) and the factor inhibiting HIF-1 (FIH) – an asparaginyl hydroxylase. PHD1-3 proteins hydroxylate two prolyl residues in the human HIF-1*α* region (oxygen-dependent degradation domain) (Epstein *et al*, 2001; Jaakkola *et al*, 2001). This HIF-*α* modification specifies rapid interaction with the von Hippel–Lindau protein, a component of an E3 ubiquitin ligase complex. Subsequently, HIF-*α* subunits become marked with polyubiquitin chains and destructed by the proteasomal system. The FIH hydroxylates an asparagine residue (N803) in the most carboxy-terminal transcriptional activation domain of human HIF-1*α*. This modification abrogates carboxy-terminal transcriptional activation domain interaction with transcriptional co-activators, such as p300/CBP. As PHDs have a much lower affinity for oxygen than FIH, PHDs will be inactivated at oxygen values that still maintain FIH activity and, therefore, keep carboxy-terminal transcriptional activation domain under repression. Thus, the two TADs, together with the oxygen-sensitive discriminator FIH, constitute a cellular device that allows fine-tuning of specific HIF gene expression along a hypoxic gradient.

Clinically only little is known on the function of the PHD3 gene with regard to cancer biology in general and pancreatic cancer growth as well (Dayan *et al*, 2006).

In human tumour specimens of pancreatic cancer PHD3 was more than 10-fold upregulated. It is interesting that the upregulation was most pronounced in tumours that were well differentiated. Undifferentiated tumours expressed lower PHD3 mRNA levels than the well-differentiated specimens. Similarly, metastatic lesions exhibited higher PHD3 mRNA levels than normal pancreatic tissue, but less than the average cancer sample, indicating a gradual loss of PHD3 mRNA during the process of tumour de-differentiation and metastasis. This finding was reproducible by *in vitro* studies using pancreatic cancer cell lines, in which well-differentiated cell lines had higher PHD3 mRNA levels than less-differentiated cell lines. *In situ* localisation of PHD3 expression was found close to neuronal invasion, but also in cancer cells. Whether this expression pattern is triggered by hypoxia only remains to be investigated in further studies as HIF-1 and CAIX did show co-localisation on serial slides, but PHD3 was not regularly detectable within this very same cellular compartment. To further address the role of PHD3 in tumour growth we applied two experimental strategies. One strategy was recombinant overexpression of PHD3 in cell lines constitutively devoid of PHD3 mRNA. The other strategy was siRNA-based downregulation of PHD3 in cell lines normally expressing PHD3 as determined qRT-PCR.

In cell culture experiments, cell growth was inhibited by increasing PHD3 levels and was accelerated by down-regulation of PHD3 expression especially under conditions of low oxygen. This effect was not due to HIF-1 activation, as the HIF-1 inhibitor YC-1 had not effect on cell growth upon PHD3 modulation. Similar findings were obtained by invasion assays, in which PHD3 exhibited an ‘anti-invasive’ phenotype under conditions of low oxygen. From cell culture experiments it was obvious that PHD3 over expression clearly changed the cell morphology with the appearance of stress fibres and floating cells in cell culture experiments. The presence of stress fibres and phosphorylated FAK is a marker of focal adhesions that anchor the F-actin filaments to transmembrane proteins and mediate communication with the cell's environment suggesting that PHD3 as oxygen sensor also influences tumour cell invasion and possibly cell migration with the biological function of escaping hypoxic areas. This effect required hydroxylase activity, but was independent of HIF-1.

Another observation was the induction of apoptosis upon PHD3 overexpression and a decreased rate of apoptosis upon PHD3 inhibition. Apparently the proapoptotic effect is mediated by caspase-3 activation, as the suppression of PHD3 decreased caspase-3 activity and over expression of PHD3 increased its activity. One remarkable observation made in human specimens was the strong immunostaining of ductal cells next to nerves within the tumour mass. Therefore, we tested the role of NGF in PHD3-mediated apoptosis. NGF withdrawal strongly suppressed apoptosis in MIA PaCa-2 and PANC-1 cells. Despite the detailed mechanism is unknown it appears that the interaction of NGF and PHD3 is of clinical interest in human specimens, as high levels of NGF close to neuronal tissue within pancreatic cancer may prevent these cells to undergo apoptosis. This observation is supported by a recent finding, in which the human PRP19 protein interacted with PHD3 to suppress cell death under hypoxic conditions by limiting the function of PHD3 (Sato *et al*, 2010).

In experimental pancreatic cancer PHD3 overexpression suppressed tumour growth in both xenografted cell lines when compared with the control groups. As PHDs represent hypoxic sensors regulating HIF-1 and VEGF *in vitro* we postulated that PHD3 overexpression and tumour growth inhibition might represent differences in the angiogenic potential. Therefore, we assayed neoangiogenesis in these xenografted tumour specimens. Assessment of intratumoural microvessel density demonstrated that PHD3 overexpression has an anti-angiogenic effect most likely by abrogating the VEGF-driven hypoxic angiogenic stimulus as observed in cell culture experiments.

In summary, we have shown that PHD3 is upregulated in human pancreatic cancer specimens with the highest average level found in well-differentiated tumours. Despite the fact that human tumours grow under conditions of low oxygen, there are most likely a variety of mechanisms rather than hypoxia causing PHD3 to be roughly 10-fold overexpressed in these specimens. Clearly, this is in contrast to the *in vitro* findings, in which PHD3 exerted tumour-protective functions as well with an increase of apoptosis and a reduced cell growth rate. Therefore, we speculate that cell growth retardation of PHD3 and induction of apoptosis are HIF-1-independent cellular effects of PHD3, whereas the anti-angiogenic activity is HIF-1 dependent. Clearly, further studies are warranted in order to address the specific functions of PHD3 in tumour growth, which may enable us to better estimate a potential benefit of therapeutic targeting of PHD3. However, the pure fact that PHD3 is highly overexpressed in human tumour specimens raises the question, whether PHD3 may represent a suitable target for therapeutic intervention against pancreatic cancer as its overexpression caused a dramatic suppression of tumour growth in experimental pancreatic cancer.
