# Supplementary figures and images for: PHD3 regulates differentiation, tumour growth and angiogenesis in pancreatic cancer
Source: Br J Cancer. 2010 Oct 26;103(10):1571–9. doi: 10.1038/sj.bjc.6605936 (PMC2990580; doi:10.1038/sj.bjc.6605936)

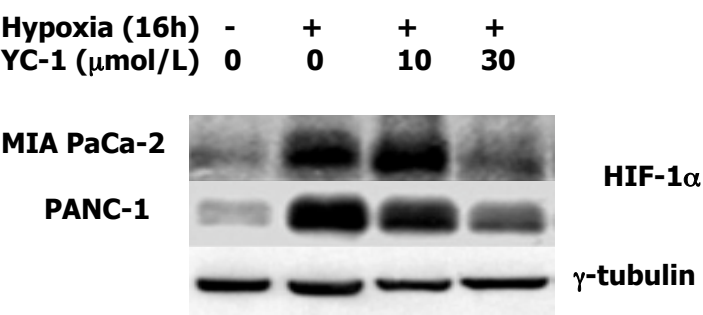

Supplement: Supplementary Figure 1 [file 6605936x1.pdf]
