# Supplementary material for: PHD3 regulates differentiation, tumour growth and angiogenesis in pancreatic cancer
Source: Br J Cancer. 2010 Oct 26;103(10):1571–9. doi: 10.1038/sj.bjc.6605936 (PMC2990580; doi:10.1038/sj.bjc.6605936)
Supplement: Supplementary Figure Legend [file 6605936x2.doc]

**Supplemental Figure:** **Dose dependent inhibition of HIF-1α upon** **YC-1 treatment:** Western blot analysis of MIA PaCa-2 and PANC-1 cells. Cells were incubated under normoxic culture condition (lane 1) or subjected for 16 hrs to hypoxic conditions (lane 2-4). YC-1 was added 5 min before hypoxic induction at doses of 10 μmol/L and 30 μmol/l.
